# Oral administration of herbal medicines for radiation pneumonitis in lung cancer patients: A systematic review and meta-analysis

**DOI:** 10.1371/journal.pone.0198015

**Published:** 2018-05-30

**Authors:** Kwan-Il Kim, Ji Hee Jun, Hyunjung Baek, Jae-Hyo Kim, Beom-Joon Lee, Hee-Jae Jung

**Affiliations:** 1 Department of Clinical Korean Medicine, College of Korean Medicine, Kyung Hee University, Seoul, Republic of Korea; 2 Division of Allergy, Immune and Respiratory System, Department of Internal Medicine, College of Korean Medicine, Kyung Hee University, Seoul, Republic of Korea; 3 Medical Research Division, Korean Institute of Oriental Medicine, Daejeon, Republic of Korea; 4 Department of Preventive Medicine, College of Korea Medicine, Daejeon University, Daejeon, Republic of Korea; 5 Department of Clinical Korean Medicine, Graduate School, Kyung Hee University, Seoul, Republic of Korea; Universita degli Studi di Napoli Federico II, ITALY

## Abstract

**Background:**

Radiation pneumonitis is a common and serious complication of radiotherapy. Many published randomized controlled studies (RCTs) reveal a growing trend of using herbal medicines as adjuvant therapy to prevent radiation pneumonitis; however, their efficacy and safety remain unexplored.

**Objective:**

The aim of this systematic review is to evaluate the efficacy and safety of herbal medicines as adjunctive therapy for the prevention of radiation pneumonitis in patients with lung cancer who undergo radiotherapy.

**Methods:**

We searched the following 11 databases: three English medical databases [MEDLINE (PubMed), EMBASE, The Cochrane Central Register of Controlled Trials (CENTRAL)], five Korean medical databases (Korean Studies Information, Research information Service System, KoreaMed, DBPIA, National Digital Science Library), and three Chinese medical databases [the China National Knowledge Database (CNKI), Journal Integration Platform (VIP), and WanFang Database]. The primary outcome was the incidence of radiation pneumonitis. The risk of bias was assessed using the Cochrane risk-of-bias tool.

**Results:**

Twenty-two RCTs involving 1819 participants were included. The methodological quality was poor for most of the studies. Meta-analysis showed that herbal medicines combined with radiotherapy significantly reduced the incidence of radiation pneumonitis (n = 1819; RR 0.53, 95% CI 0.45–0.63, I^2^ = 8%) and the incidence of severe radiation pneumonitis (*n* = 903; RR 0.22, 95% CI 0.11–0.41, I^2^ = 0%). Combined therapy also improved the Karnofsky performance score (n = 420; WMD 4.62, 95% CI 1.05–8.18, I^2^ = 82%).

**Conclusion:**

There is some encouraging evidence that oral administration of herbal medicines combined with radiotherapy may benefit patients with lung cancer by preventing or minimizing radiation pneumonitis. However, due to the poor methodological quality of the identified studies, definitive conclusion could not be drawn. To confirm the merits of this approach, further rigorously designed large scale trials are warranted.

## Introduction

Lung cancer is the most common type of cancer in both men and women, and one of the main causes of cancer death worldwide. In 2016, 243,820 new cases of lung cancer were expected, and estimated to comprise 27% of all male cancer deaths and 26% of female cancer deaths [1]. In Korea, lung cancer mortality rates in 2013 were 34.0% for both sexes, accounting for 49.5% of all male cancer deaths and 18.4% of all female cancer deaths [2].

Radiotherapy has been widely used for unresectable and locally advanced non-small cell lung cancer (NSCLC) and small-cell lung cancer (SCLC) [3, 4]. However, the lung is more vulnerable to radiotherapy than other organs, and radiation leads to pulmonary toxicity [5, 6]. Radiotherapy pneumonitis (RP) caused by radiation-induced lung toxicity is the most serious complication [7, 8]. RP typically presents 1–6 months after radiation therapy [6, 9, 10]. The clinical features usually include mild dry cough, mild fever, and mild dyspnea, but in some cases, severe respiratory failure appears and leads to death [6, 8, 9]. The incidence of moderate to severe RP with radiotherapy is 10–20%, but varies in clinical studies [8, 11]. When RP is left untreated for a long time, it may develop into pulmonary fibrosis, which has a high rate of mortality. The development of RP sometimes makes cessation of radiotherapy or control of the radiation dose necessary. RP not only decreases the treatment success rate for lung cancer, but also reduces the patient’s quality of life. Therefore, it is very important to prevent or minimize the incidence of RP.

Despite great efforts to develop agents to reduce the severity and incidence of pulmonary toxicity resulting from radiotherapy, no effective agents currently exist [12]. Amifostine has broad applicability as a protective agent, but the results of clinical trials are controversial. The reported results have not been replicated, and present guidelines and systematic reviews do not support the use of amifostine for the prevention of RP [12, 13].

Herbal medicines (HMs) are commonly used complementary and alternative therapies in cancer treatment [14]. Lung cancer patients also use HM while receiving chemotherapy or radiotherapy. A study reported that chemotherapy combined with administration of HMs increased the survival rate among patients with lung cancer [15]. A recent systematic review reported that *Astragalus*-containing HMs are effective at protecting against RP as adjunctive therapy during conservative radiotherapy [16]. There are also many published trials of HMs other than *Astragalus* that examined protective effects against RP, but the evidence for HMs protecting against or minimizing RP is not yet compelling. Therefore, we sought to carry out a comprehensive systematic review of the efficacy and safety of HM as adjunctive therapy to prevent RP in lung cancer patients receiving radiotherapy.

## Methods

This study was registered with the international Prospective Register of Systematic Reviews (PROSPERO): CRD 42016048066 and the protocol was published [17].

### Selection criteria

#### Types of studies and participants

Only randomized controlled trials (RCTs) were included. Crossover studies, observation studies and case studies were excluded. Patients were included if they were diagnosed with lung cancer, aged 18 or older, and planned to undergo radiotherapy, regardless of tumor stage.

#### Type of interventions

Studies reporting orally administered HM treatment as adjunctive therapy with radiotherapy were included regardless of the HM type. HMs refers to a treatment involving single herb or a combination of herbs. Studies that included other alternative and complementary therapies, such as acupuncture, moxibustion, massage, etc. were excluded. Trials using control groups receiving radiotherapy or radiotherapy combined with placebo control were included. We only excluded stereotactic radiation therapy among radiation techniques based on Radiation oncologist’s opinion. Trials involving other types of therapy, including chemotherapy, were excluded.

#### Type of outcome measures

Primary outcomes: The rate of incidence f RP after radiotherapy was analyzed as the primary outcome. Trials that reported the diagnostic criteria for RP in terms of commonly used grading systems, such as the National Cancer Institute Common Terminology Criteria (NCICTC) for Adverse Events, the Radiation Therapy Oncology Group (RTOG) score, or the Common Terminology Criteria for Adverse Events (CTCAE) were included [18]. Studies using clinical criteria for RP, in which the diagnosis was made based on the patient’s symptoms (including shortness of breath, intermittent low fever, cough, congestion, etc.) combined with radiological manifestations [19] were also included.

Secondary outcomes: The secondary outcome assessments consisted of the Karnofsky performance status (KPS) score, pulmonary function test—especially the diffusing capacity of the lungs for carbon monoxide (DLCO)—and adverse events.

### Search methods for the identification of studies

We searched the following databases: three English medical databases (PubMed, EMBASE, The Cochrane Library), five Korean medical databases (Korean Studies Information, Research information Service System, KoreaMed, DBPIA, National Digital Science Library), and three Chinese medical databases (the China National Knowledge Database (CNKI), Journal Integration Platform (VIP), and WanFang Database). Related gray literature and references of included studies were hand searched. The databases were searched from their inceptions up to July 2017. The studies were not limited by language.

The key search terms were “radiation pneumonitis” and “herbal medicine.” We used related Medical Subject Heading terms and synonyms in various combinations. The search strategy is presented in online S1 Appendix.

### Data collection and analysis

#### Selection of studies

Two reviewers (KIK and JHJ) selected eligible studies according to the inclusion criteria. The studies were screened based on the study design, patients, intervention, comparator, and outcome, derived from the title and abstract. The full texts of the surviving studies were then reviewed independently for inclusion in the study. Discrepancies were resolved by discussion with a third researcher (HJJ), to determine, by agreement, the final selection of studies.

#### Data extraction and management

Data were independently extracted by two reviewers (KIK and BJL). Data including publication year, country, inclusion/exclusion criteria, lung cancer type (NSCLC, SCLC), tumor stage, diagnosis criteria for RP, patient’s age, sex, interventions (composition, dosage, and type of the intervention, treatment duration), comparator, outcomes, adverse events, radiation dosage, randomization, blinding, and number of withdrawals and dropouts were recorded in an Excel spreadsheet. Any discrepancies in the data were resolved by discussion with another team member (HJJ).

### Risk of bias assessment

Three reviewers (KIK, JHJ and BJL) independently assessed the risk of bias for each included study, according to section 5.1 of the Cochrane Handbook [20]. The following criteria were used: (1) random sequence generation, (2) allocation concealment, (3) blinding of participants and personnel, (4) blinding of outcome assessment, (5) incomplete outcome data, (6) selective outcome reporting, and (7) other sources of bias (baseline imbalance). We used “L,” “H,” and “U” as a code for the judgments, “L” indicating a low risk of bias, “H” indicating a high risk of bias, “U” indicating that the risk of bias was unclear.

Disagreements were resolved by discussion between all the reviewers. When necessary, we contacted the study authors to clarify protocols and obtain any missing information.

### Data synthesis

Review Manager Software (RevMan, Version 5.3 for windows; Copenhagen, The Nordic Cochrane Centre, The Cochrane Collaboration, 2014) was used for data analysis. For dichotomous outcomes, we used the risk ratio, with 95% CI and p values, to assess the efficacy and safety of HM. For continuous data collected using the same measurement scale, we calculated the weighted mean difference (WMD) and 95% confidence intervals (CIs). A Chi-square test with a significance level of p < 0.1 was used to assess heterogeneity among studies. To assess inconsistencies among studies, the I^2^ test was used. The I^2^ statistic indicates the proportion of variability among studies not explained by chance alone, and an I^2^ value > 50% or more is considered an indication of substantial heterogeneity. If heterogeneity existed in the pooled studies, a random effects model was applied. Otherwise, the data were analyzed using a fixed-effect model. Funnel plots were used to detect publication bias when 10 or more studies were included in a meta-analysis.

### Assessment of reporting bias

A funnel plot and Egger’s test will be used to detect publication bias when 10 or more studies are included in the meta-analysis.

## Results

### Characteristics of the included studies

**Fig 1:**
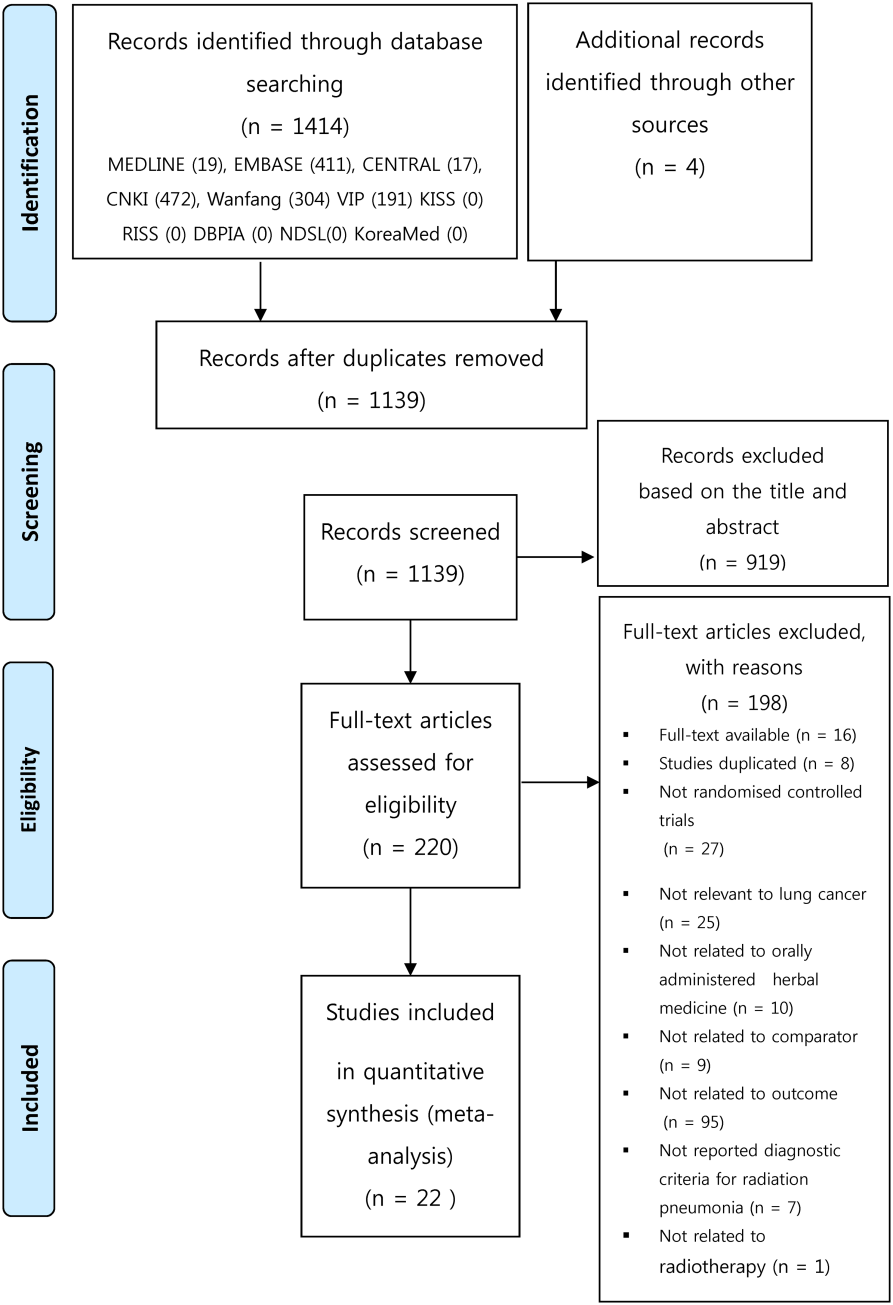
Flow diagram of literature search.

In this study, a total of 1418 studies were retrieved through electronic and manual searches. Duplicate studies were excluded. During the first screening process, 919 studies were excluded based on their titles and abstracts. After reviewing the full texts of 220 studies, 198 studies were excluded, and the remaining 22 studies [21–42] were finally included in this study. The reference of exclude studies in S2 Appendix. The study selection process details are described in Fig 1. A total of 1819 subjects were included in the analysis. Twenty-four subjects withdrew or dropped out.

All the included studies were published in China. There were 17 studies conducted on patients with NSCLC [21–25, 30, 31, 33–42], and 4 studies conducted on both patients with NSCLC and patients with SCLC [26, 27, 29, 32]. The types of HMs that were orally administered were as follows: herbal decoctions were used in 15 studies [21–23, 25–29, 32, 33, 35–37, 39, 40], capsules were used in 4 studies [24, 38, 41, 42], pills were used in 2 studies [31, 34], and a granule was used once [30]. The duration of HM treatment varied from 4 weeks to 28 weeks. Only 3 studies used a treatment duration greater than 12 weeks [22, 29, 42]. Conventional radiotherapy was applied in 9 studies [21, 23–25, 27–28, 31, 34, 39]; 9 studies [22, 26, 30, 33, 35–38, 41] employed three-dimensional conformal radiotherapy; intensity-modulated radiotherapy were adopted in 2 studies [40, 42]; and 2 studies [29, 32] employed three-dimensional conformal radiotherapy or intensity-modulated radiotherapy. The dose of radiation therapy varied from 30 to 70 Gy in the included studies. The detailed characteristics of the eligible studies are shown in Table 1.

**Table 1:** Characteristics of the included studies.

First author (year)	Patients (drop-out) No.; Age	Lung cancer type / TNM stage	Intervention group	Control group	Type of RT Dose of RT	Outcome	Results
Wang (2006)	85 (A) 61.3 (B) 60.7	NSCLC / n.r.	(A) HM (Jiawei Baihe Gujin decoction, 2 times a day for 7 weeks) + (B), n = 48	(B) Radiotherapy, n = 37	CRT 70 Gy (2 Gy/f)	1) The incidence rate of RP 2) RP incidence (RTOG>3)	1) RR 0.30 [0.12, 0.76] 2) RR 0.22 [0.05, 1.00]
Zhang (2006)	74 (5) (A) 56.5 (B) 59.5	NSCLC / III	(A) HM (HM decoction (LC1, LC2), LC1 for 4 weeks, after 1-week rest, LC2 for.24 weeks) + (B), n = 32	(B) Radiotherapy, n = 37	3D-CRT 55–62 Gy (4–5 Gy/f)-	1) The incidence rate of RP 2) RP incidence (RTOG>3)	1) RR 0.87 [0.21, 3.59] 2) No event
Song (2007)	120 (5) (A) 60.8±5.7 (B) 60.7±5.2	NSCLC / III	(A) HM (Zengye decoction, 2 times a day for 4 weeks) + (B), n = 57	(B) Radiotherapy, n = 58	CRT 60–70 Gy	1) The incidence rate of RP	1) RR 0.85 [0.58, 1.25]
Fu (2008)	148 Total median 74 (70–83)	NSCLC / III-IV	(A) HM (Zhenqi Fuzheng Capsules, 3 times a day for 7 weeks) + (B), n = 74	(B) Radiotherapy, n = 74	CRT 60–70 Gy (2 Gy/f)	1) The incidence rate of RP	1) RR 0.41 [0.22, 0.76]
Tang (2009)	60 (A) 62.1±7.94 (B) 58.9±9.71	NSCLC / I-III	(A) HM (Yiqi Giedu decoction, 2 times a day for 6~7 weeks) + (B), n = 30	(B) Radiotherapy, n = 30	CRT 60–70 Gy (1.5–1.8 Gy/f)	1) The incidence rate of RP 2) KPS	1) RR 0.50 [0.25, 0.99] 2) MD 11.00 [6.30, 15.70]
Zhou (2009)	60 Total median 58 (36–82)	NSCLC, SCLC/ n.r.	(A) HM (Yiqi Yangyin Gingfei decoction, 2 times a day for 8 weeks) + (B), n = 30	(B) Radiotherapy, n = 30	3D-CRT 1) NSCLC: 60–66 Gy 2) SCLC: 46–50 Gy	1) The incidence rate of RP	1) RR 0.42 [0.17, 1.04]
Xiao (2010)	100 (12) (A) 55.66±15.38 (B) 59.32±10.12	NSCLC, SCLC / I-IV	(A) HM (Liangxue Jiedu Huoxue decoction, 2 times a day for 8 weeks) + (B), n = 46	(B) Radiotherapy, n = 42	CRT 30–40 Gy	1) The incidence rate of RP 2) KPS	1) RR 0.39 [0.17, 0.92] 2) MD 4.98 [1.16, 8.80]
Jiang (2011)	86 (A) 56.8±7.1 (B) 58.2±6.3	n.r. / n.r.	(A) HM (Maxuean Zhike decoction, 3 times a day for 4weeks) + (B), n = 42	(B) Radiotherapy, n = 44	CRT 50–70 Gy (2 Gy/f)	1) The incidence rate of RP 2) RP incidence (RTOG>3)	1) RR 0.45 [0.19, 1.06] 2) No event
Wang (2011)	83 Total median 56 (33~83)	NSCLC, SCLC / I-III	(A) HM (Zhongfei decoction, 3 times a day for 9~19weeks) + (B), n = 40	(B) Radiotherapy, n = 41	3D-CRT or IMRT 45–70 Gy 1) NSCLC 60–70 Gy 2) SCLC 50–60 Gy	1) The incidence rate of RP 2) RP incidence (RTOG>3)	1) RR 0.80 [0.37, 1.72] 2) RR 0.22 [0.05, 0.94]
Gao (2012)	158 (A) 70~81 (B) 67~85	NSCLC / I-IV	(A) HM (Shenqi ten granules, 3 times a day for 8weeks) + (B), n = 79	(B) Radiotherapy, n = 79	3D-CRT 60–66 Gy (1.8Gy/f)	1) The incidence rate of RP 2) RP incidence (RTOG>3)	1) RR 0.33 [0.14, 0.80] 2) RR 0.33 [0.07, 1.60]
Meng (2012)	78 (A) 58.59±11.67 (B) 54.23±13.15	NSCLC / III	(A) HM (Maiwei Dihuang Wan, 3 times a day for 12weeks) + (B), n = 40	(B) Radiotherapy, n = 38	CRT 40-70Gy	1) The incidence rate of RP 2) RP incidence (RTOG>3) 3) KPS	1) RR 0.44 [0.19, 1.04] 2) RR 0.19 [0.02, 1.55] 3) MD -4.00 [-7.76, -0.24]
Cao (2013)	70 (A) 57.12 ± 7.15 (B) 58.92 ± 6.55	NSCLC, SCLC / n.r.	(A) HM (HM decoction, 2 times a day for 10–12 weeks) + (B), n = 35	(B) Radiotherapy, n = 35	3D-CRT or IMRT 45–70 Gy 1) NSCLC 60-70Gy 2) SCLC 50–60 Gy	1) The incidence rate of RP 2) RP incidence (RTOG>3)	1) RR 0.67 [0.27, 1.67] 2) RR 0.25 [0.06, 1.09]
Du (2013)	40 (A) 60.85 ±7.22 (B) 60.35 ±6.95	NSCLC / II-,III	(A) HM (Jingtian Fuzhang Kangai HM decoction, 2 times a day for 6~7weeks) + (B), n = 20	(B) Radiotherapy, n = 20	3D-CRT 60–70 Gy (2 Gy/f)	1) The incidence rate of RP 2) RP incidence (RTOG>3) 3) KPS	1) RR 0.40 [0.15, 1.07] 2) RR 0.33 [0.01, 7.72] 3) MD 6.00 [0.73, 11.27]
Xu (2013)	39 (A) 62.30 ± 7.53 (B) 63.10 ± 7.36	NSCLC / III	(A) HM (Feifukang pill, 3 times a day for 12weeks) + (B), n = 20	(B) Radiotherapy, n = 19	CRT 3–7 Gy/f total 12–14 times	1) The incidence rate of RP	1) RR 0.38 [0.08, 1.73]
Yin (2013)	78 Total 55–82	NSCLC / III	(A) HM (HM decoction, 2 times a day for 6–7 weeks) + (B), n = 40	(B Radiotherapy, n = 38	3D-CRT 60–66 Gy (2 Gy/f)	1) The incidence rate of RP	1) RR 0.53 [0.28, 0.99]
Li (2014)	96 (A) 57.3 ± 7.1 (B) 58.6	NSCLC / I-IV	(A) HM (Yiqi Yangyin decoction, 2 times a day for 8weeks) + (B), n = 48	(B) Radiotherapy, n = 48	3D-CRT 54–60 Gy (1.8–2.0 Gy/f)	1) The incidence rate of RP 2) RP incidence (RTOG>3) 3) KPS	1) RR 0.53 [0.25, 1.14] 2) RR 0.33 [0.01, 7.98] 3) MD 5.00 [3.00, 7.00]
Lu (2016)	80 (A) median 63.4 (B) median 65.2	NSCLC / III	(A) HM (Fuzheng decoction, 2~3 times a day for 6 weeks) + (B), n = 43	(B) Radiotherapy, n = 37	3D-CRT 54–60 Gy (1.8–2.0 Gy/f)	1) The incidence rate of RP 2) RP incidence (RTOG>3)	1) RR 0.48 [0.25, 0.90] 2) RR 0.06 [0.00, 0.98]
Wang (2016)	72 Total median 66 (60–73)	NSCLC / III	(A) HM (Yangzhengxiaoji capsule, 3times a day for 6–6.5 weeks), n = 36	(B) Radiotherapy, n = 36	3D-CRT 60–66 Gy (2 Gy/f)	1) The incidence rate of RP	1) RR 0.67 [0.46, 0.97]
Xie (2016)	60 (2) (A) 54.1±5.1 (B) 53.8±4.5	NSCLC / III	(A) HM (Fuzheng Jiandu Kangai decoction, 2 times a day for 4 weeks), n = 30	(B) Radiotherapy, n = 28	CRT 60–70 Gy	1) The incidence rate of RP 2) KPS	1) RR 0.31 [0.09, 1.03] 2) MD 5.53 [1.35, 9.71]
Zhang (2016)	56 (A) 66.4± 5.2 (B) 65.9± 5.5	NSCLC / II	(A) HM (Jiawei Baihe Gujin decoction + Biyan qing du Keli, 2 times a day for 2 weeks) + (B), n = 28	(B) Radiotherapy, n = 28	IMRT 50–60 Gy	1) The incidence rate of RP	1) RR 0.10 [0.03, 0.39]
Sun (2017)	120 (A) 36–69 (B) 34–69	NSCLC / II-III	(A) HM (Xufu zhuyu capsule, 2 times a day for 12 weeks) + (B), n = 60	(B) Radiotherapy, n = 60	3D-CRT 50–60 Gy	1) The incidence rate of RP	1) RR 0.86 [0.31, 2.40]
Zhang (2017)	80 n.r.	NSCLC / II-III	(A) HM (Xihuang capsule, 2 times a day for 24 weeks) + (B), n = 40	(B) Radiotherapy, n = 40	IMRT 50–60 Gy	1) The incidence rate of RP	1) RR 0.36 [0.13, 1.05]

HM: herbal medicine; RTOG: radiation therapy oncology group; KPS: karnofsky performance status scale; NSCLC: non small cell lung cancer; SCLC: small cell lung cancer; n.r.: not reported, RT: radiotherapy, CRT: conventional radiotherapy, IMRT: intensity-modulated radiotherapy, 3D-CRT: three-dimensional conformal radiotherapy.

### Risk of bias assessment of the included studies

The risk of bias for each study was assessed according to section 5.1.0 of the Cochrane Handbook for Systematic Reviews of Interventions. Seven studies described the method of randomization, and six of them reported using the random number table method, hence were evaluated as “low” [25, 32, 36, 37, 39, 42]. One study used patient medical record numbers, and was evaluated as “high” [33]. The other studies did not report any randomization procedure, and were evaluated as “unclear.” The allocation procedure was not reported for any of the studies, so we evaluated all of them as “unclear.”

**Fig 2:**
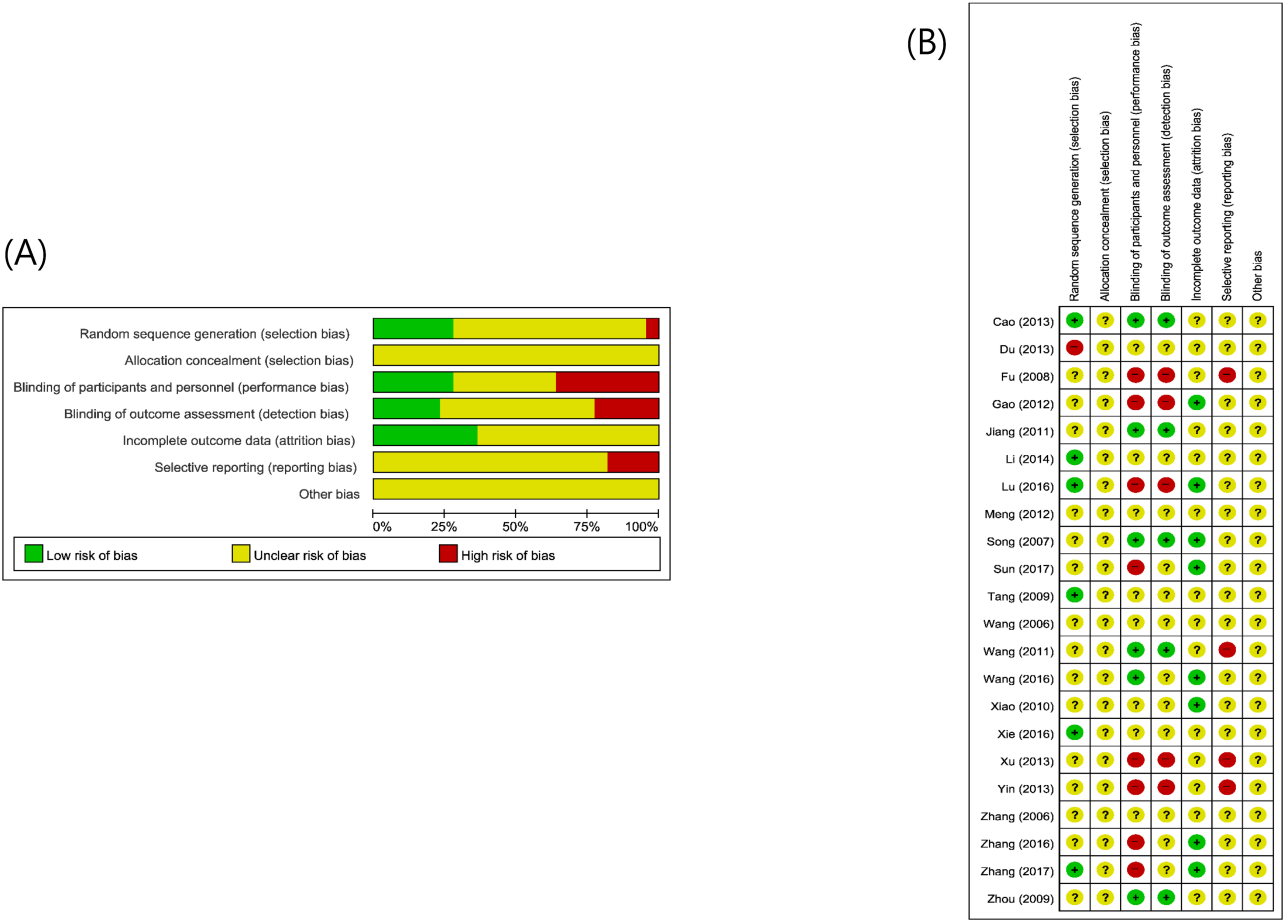
Risk of bias. (A) Risk of bias graph: review authors’ judgments about each item’s risk of bias item presented as percentage across all included studies. (B) Risk of bias summary: review authors’ judgments about each item’s risk of bias for each included study. +: low risk of bias; −: high risk of bias;?: unclear.

Since placebos were not used in any of the studies, blinding was not performed in any of them. However, when the primary outcome was the RP incidence rate, it was considered that clinical judgements about the occurrence of RP would not always be affected by blinding. Therefore, studies that clearly described the diagnostic criteria for RP, and confirmed RP on X-ray or CT scans, were evaluated as “low” However, studies in which subjective assessment parameters were included in the secondary outcomes, making estimation of the influence of blinding on the study results difficult, were evaluated as “unclear.” Studies that did not include objective diagnostic tools such as medical imaging findings in the diagnostic criteria for RP, and performed subjective assessment, were evaluated as “high,” since blinding could have affected the study results. Studies with incomplete outcome data were evaluated as “low” if they reported missing data and reasons for the missing data, and it was judged that the missing data did not affect the study results. Studies that did not report missing data were evaluated as “unclear.” With regard to selective bias, four studies that did not present evaluation results for the outcome parameters were evaluated as “high.” The remaining studies were evaluated as “unclear,” since assessment of selective bias in these studies was not possible due to insufficient information. With regard to other bias, all studies were evaluated as “unclear,” since full assessment of bias in these studies was not possible due to insufficient information (Fig 2).

### Outcome

#### Incidence rate of RP

**Fig 3:**
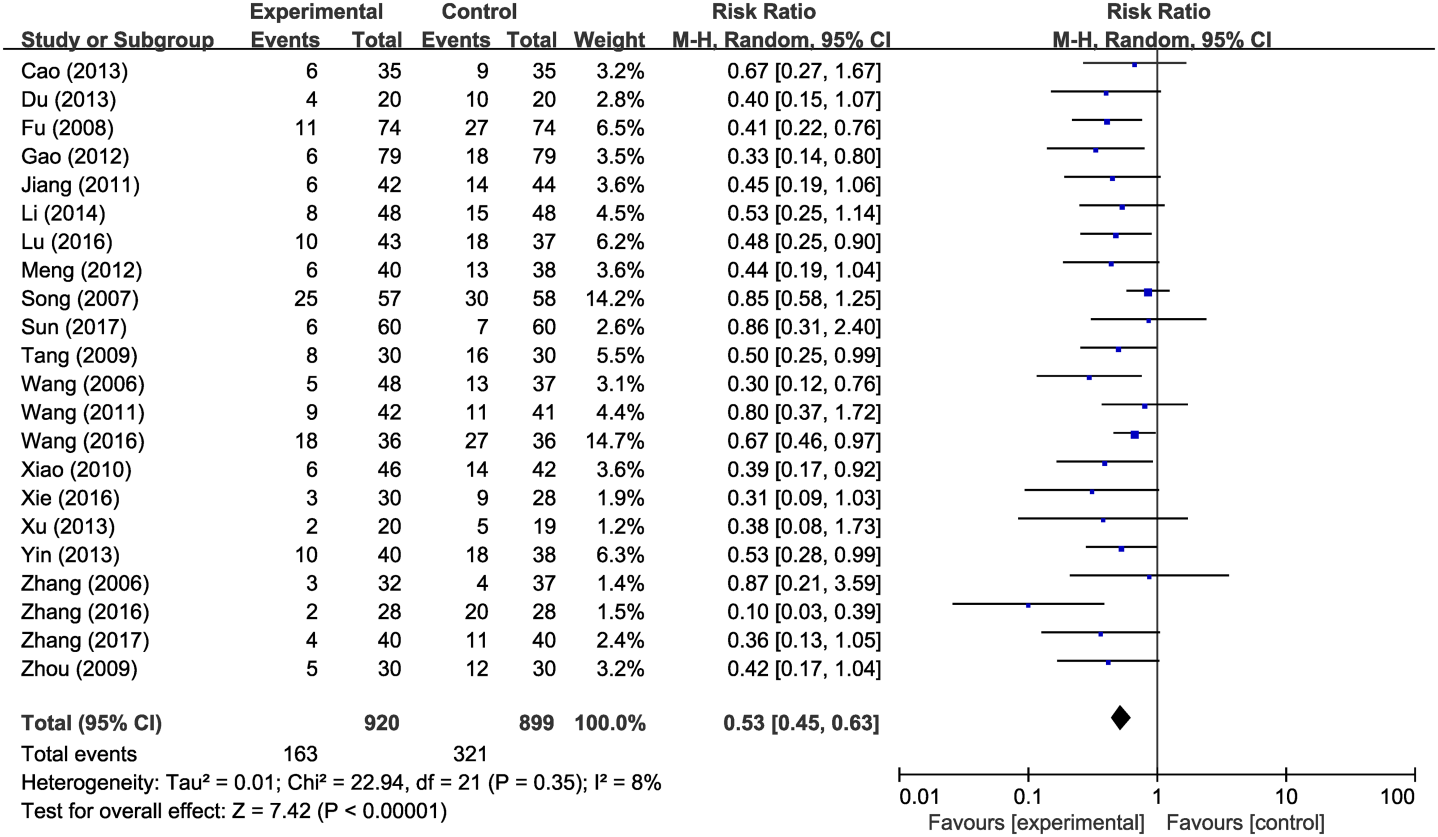
Forest plot of incidence of radiation pneumonitis.

**Fig 4:**
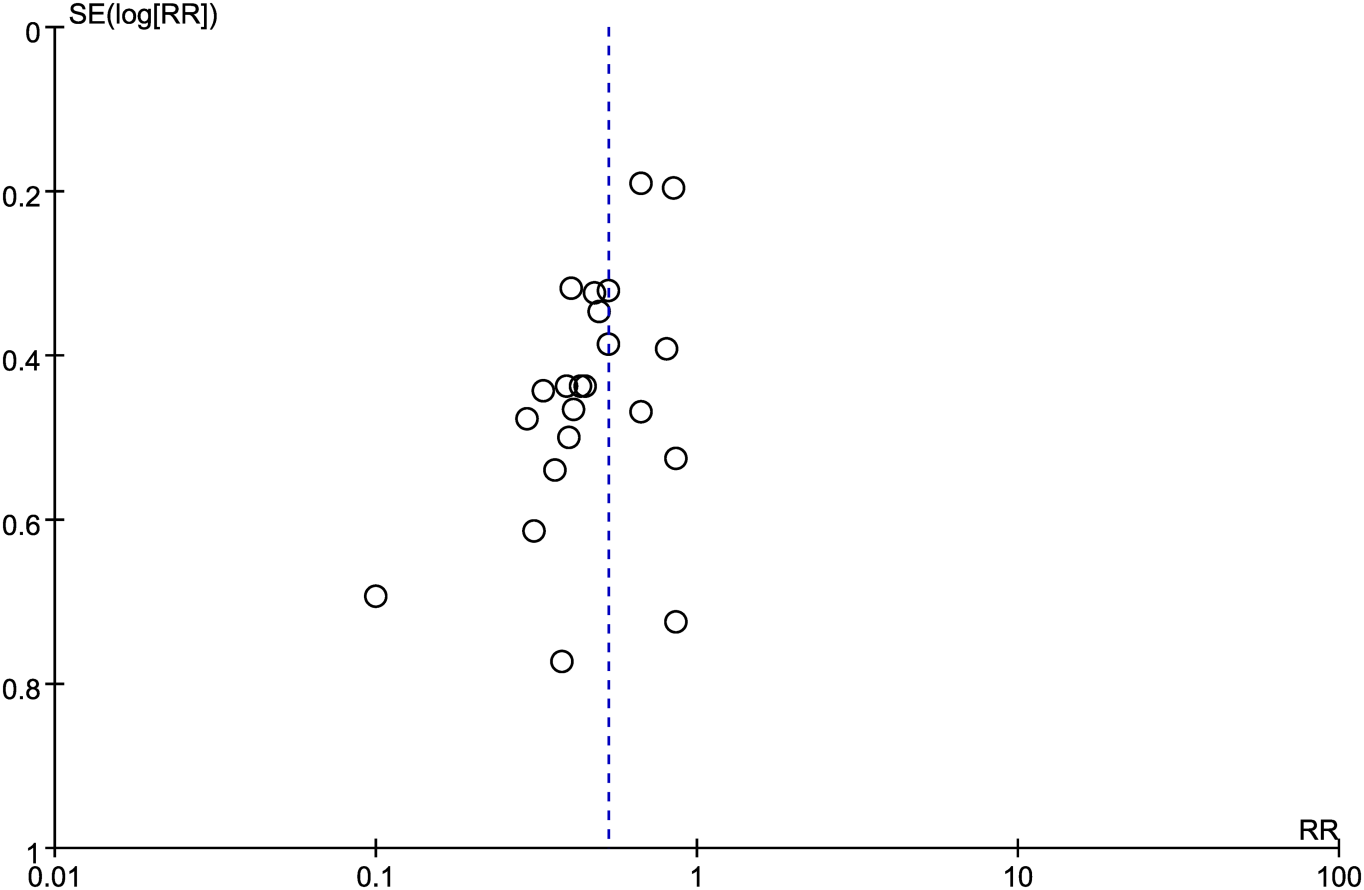
Funnel plot of studies testing for incidence of radiation pneumonitis.

We analyzed the incidence rates of RP among patients who were administered HM during radiotherapy and patients who underwent radiotherapy alone, in 22 studies conducted on a total of 1819 clinical trial participants [21–42]. The relative risk (RR) of RP was significantly less than 1 in a comparison between the HM plus radiotherapy group and the radiotherapy alone group (RR 0.53; 95% CI, 0.45–0.63) (Fig 3). However, an asymmetric funnel plot was observed, suggesting the existence of publication bias (Fig 4).

**Fig 5:**
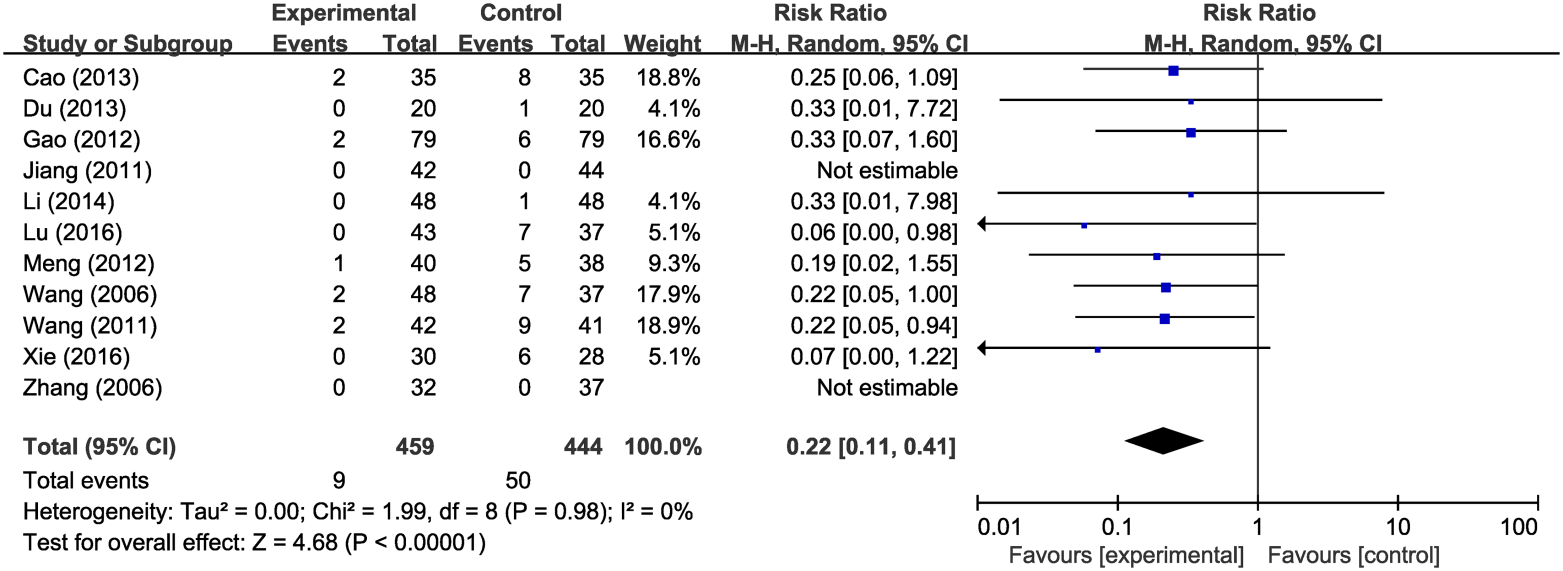
Forest plot of incidence of severe radiation pneumonitis.

We also analyzed the incidence rate of severe radiation pneumonitis, scoring 3 or more on the RTOG scale. A total of 11 studies, involving 903 patients, were included in the analysis [21, 22, 28–33, 36, 37, 39]. The analysis result showed that the RR of radiation pneumonitis was significantly less than 1 in the HM plus radiotherapy group compared to the radiotherapy alone group (RR 0.22; 95% CI, 0.11–0.41) (Fig 5).

#### Effects on quality of life (QoL)

**Fig 6:**
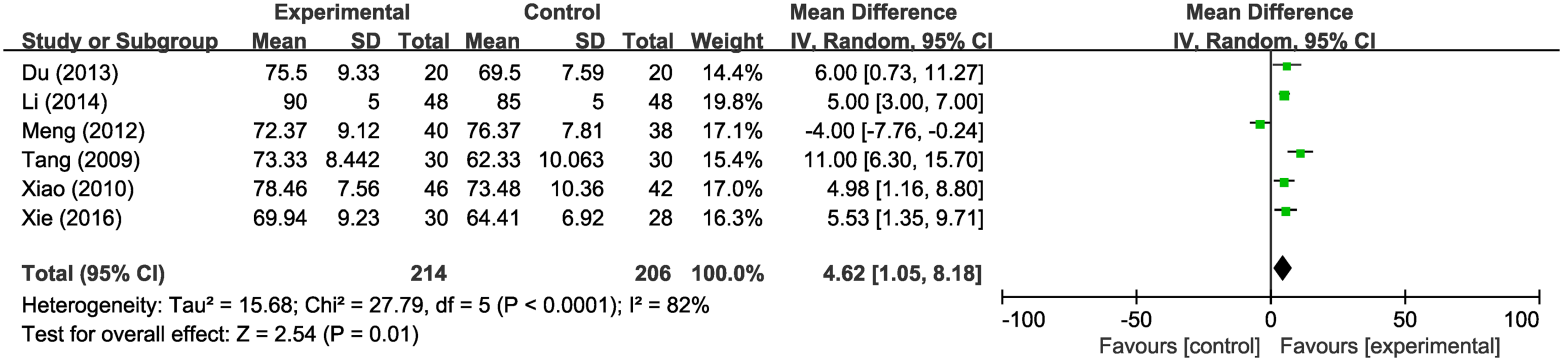
Forest plot of quality of life.

As can be seen in Fig 6, six studies (involving 420 patients) that reported the KPS as mean and standard deviation were included [25, 27, 31, 33, 36, 39]. The HM plus radiotherapy group showed significantly better performance status than the radiotherapy alone group (WMD 4.62, 95% CI 1.05–8.18). Only six trials reported performance status, so a funnel plot was not applicable.

#### Effects on lung function

One study was identified, including 78 patients that reported lung functions [35]. The forced vital capacities (FVC) was 2.44±0.40 in the HM plus radiotherapy group and 2.28±0.28 in the radiotherapy alone group. The forced expiratory volumes in 1 second (FEV1) was 2.02±0.34 in the HM plus radiotherapy group and 1.83±0.40 in the radiotherapy alone group. The diffusing capacity of the lungs for carbon monoxide (DCLO) was 13.36±3.96 in the HM plus radiotherapy group and 11.22±2.88 in the radiotherapy alone group. The differences in all these lung functions between the two groups were statistically significant (p<0.05).

#### Adverse events (AEs)

Four trials investigated the side effects of HMs [25–27, 31]. Of these, three studies reported no side effects in either the HM plus radiotherapy group or the radiotherapy alone group [25, 26, 31]. One study reported mild diarrhea in two patients from the HM plus radiotherapy group [27]. For assessment of the safety of HMs, liver function tests (LFT) and renal function tests (RFT) were performed in these four studies. No abnormal findings were identified.

#### Herbal composition and frequency

Twenty-two studies reported the herbal formula, in the form of a decoction, capsules, granules, etc. The herbal formulas used in combination with radiation therapy consisted mainly of those that tonify yin, tonify qi, and nourish blood. The detailed herbal compositions are shown in Table 2. Among them, *Ophiopogonis Radix* was the most commonly used herb for lung cancer as adjunctive therapy with radiotherapy. *Ophiopogonis Radix*, *Adenophorae Radix* and *Reheanniae Radix Praeparata* are also well-known herbs that nourish yin. *Astragali Radix* is a typical herb with qi-tonifying effects, and *Angelicae Sinensis Radix* is a classic herb that nourishes blood (Table 3).

**Table 2:** Compositions of the included herbal formula.

First author (years)	Herbal formula	Main composition of formula	Matching composition of formula
**Wang (2006)**	Jiawei Baihe Gujin decoction	Angelicae Gigantis Radix 9g, Astragali Radix 12g, Coicis Semen 15g, Fructus ligustri lucidi 12g, Glycyrrhizae Radix et Rhizoma 6g, Lilii Bulbus 9g, Liriopis Tuber 9g, Rehmanniae Radix Crudus 12g, Rehmanniae Radix Paeoniae Radix 6g, Preparata 12g,Pseudostellariae Radix 12g, Platycodi Radix 9g, Scrophulariae Radix 9g, the bulb of fritillary 9g, Scutellariae Barbatae Herba 15g,	
**Zhang (2006)**	HM decoction (LC1, LC2)	Angelicae Sinensis Radix, Astragalus, Coicis Semen, Fritillariae Cirrhosae Bulbus, Glycyrrhizae Radix et Rhizoma, Lilii Bulbus, Ophiopogonis Radix, Paeoniae Radix Alba, Platycodonis Radix, Pseudostellariae Radix, Rehmanniae Radix Preparata, Scrophulariae Radix, Scutellariae Barbatae Herba	
**Song (2007)**	Zengye decoction	Ginseng Radix et Rhizoma, Glenniae Radix, Ophiopogonis Radix, Rehmanniae Radix	
**Fu (2008)**	Zhenqi Fuzheng Capsules	Ligustri lucidi Fructus, Stragali radix	
**Tang (2009)**	Yiqi Giedu decoction	Angelicae sinensis Radix, Astragali Radix, Cyperi Rhizoma, Lycii Fructus, Pheretima, Polygoni Cuspidati Rhizoma et Radix, Rehmanniae Radix, Schisandrae Fructus, Spatholobi Caulis	
**Zhou (2009)**	Yiqi Yangyin Gingfei decoction	Adenophorae Radix, Amarum Trichosanthis Fructus, Ardisiae Herba, Armeniacae Semen, Astragali Radix, Eriobotryae Folium, Glenniae Radix, Ligustri lucidi Fructus, Mori Cortex, Ophiopogonis Radix, Poria Atractylodis macrocephalae Rhizoma, Pseudostellariae Radix, Sophorae flavescentis Radix, Spatholobi Caulis, Stemonae Radix	
**Xiao (2010)**	Liangxue Jiedu Huoxue decoction	Astragali Radix, Carthami Flos, Chuanxiong Rhizoma, Forsythiae Fructus, Moutan Cortex, Persicae Semen, Rehmanniae Radix	
**Jiang (2011)**	Maxuan Zhike decoction	Amarum Gypsum Fibrosum, Armeniacae Semen, Belamcandae Rhizoma, Ephedrae Herba, Glycyrrhizae Radix et Rhizoma, Houttuyniae Herba, Lonicerae japonicae Flos, Ophiopogonis Radix, Platycodonis Radix, Scrophulariae Radix	
**Wang (2011)**	Zhongfei decoction	n.r.	
**Gao (2012)**	Shenqi ten granules	Alismatis Rhizoma, Angelicae Sinensis Radix, Asari Radix et Rhizoma, Astragali Radix, Cassiae Semen, Cervi Cornu, Cuscutae Semen, Gastrodiae Rhizoma, Ginseng Radix et Rhizoma, Lycii Fructus, Rehmanniae Radix Praeparata	
**Meng (2012)**	Maiwei Dihuang pills	Alismatis Rhizoma, Alismatis Rhizoma, Corni Fructus Tostum, Dioscoreae Rhizoma, Moutan Cortex, Ophiopogonis Radix, Poria, Rehmanniae Radix Preparata, Schisandrae Chinesis Fructus	
**Cao (2013)**	HM decoction	Angelicae Sinensis Radix 10g, Atractμlodis Macrocephalae Rhizoma 10g, Chiysanthmi Indici Flos 15G, Chrysanthemi Indici Flos 15g, Cuscutae Semen 10g, Glehniae Radix 15g, Hedyotis Diffusa Willd30g, Ligustri Lucidi Fructus 10g, Liriopes Radix 10g, Lonicerae Japonicae Flos 15g, Lonicerae Japonicae Flos 15g, Lycii Fructus 10g, Poria 10g, Schisandrae Chinensis Fructus 6g, Scutellariae Radix 15g, Semiaquilegiae Radix 15g, Semiaquilegiae Radix 15g, Taraxaci Herba 15g, Taraxaci Herba 15g, Violae Herba 15g, Violae Herba 15g	much yellowish sputum, add Arisaema Cum Bile 10g, Trichosanthis Pericarpium 12g; severe cough, addPrunus Armeniaca L.10g, Farfarae Flos 10g; bloody sputum, add Agrimoniae Herba 30g, Typhae Pollen Carbonisata 10g
**Du (2013)**	Jingtian Fuzhang Kangai HM decoction	Adenophorae Radix 20g, Angelicae Sinensis Radix 15g, Astmgali Radix 30g, Aurantii Fructus 10g, Chebulae Fructus Immaturus 10g, Corni Fructus 10g, Curcumae Rhizoma 10g, Cyperi Rhizoma 10g, Fritillariae Thunbergii Bulbus 10g, Glycyrrhizae Radix et Rhizoma 6g, Herba Salviae Chinensis 20g, Massa Medicata Fermentata 10g, Mori Fructus 30g, Polygonati Rhizoma 10g, Rhodiolae Crenulatae Radix et Rhizoma 15g, Sparganii Rhizoma 10g, Spatholobi Caulis 20g, Trionycis Carapax 30g	
**Xu (2013)**	Feifukang pill	Bletillae Rhizoma, Descurainiae Semen Lepidii Semen, Fritillariae Thunbergii Bulbus, Gecko, Ginseng Radix et Rhizoma Rubra, Hominis Placenta, Pheretima, Stemonae Radixpraeparata Cum Melle	
**Yin (2013)**	HM decoction	Arnebiae Radix 15g, Chuanxiong Rhzoma 10g, Dendrobii Caulis 20g, Glycyrrhizae Radix et Rhizoma 10g, Houttuyniae Herba 20g, Moutan Cortex 20, Ophiopogonis Radix 20g, Paris Polyphylla 15g, Patriniae Herba 15g, Pini Pollen or Trichosanthis Radix 20g, Radix et Rhizoma 15g	
**Li (2014)**	Yiqi Yangyin decoction	Adenophorae Radix 30g, Atractylodis Rhizoma Alba 15g, Codonopsis Pilosulae Radix 30g, Liriopis Tuber 30g, Lilii Bulbus 15g, Paeoniae Radix15g, Rehmanniae Radix Preparata 30g, Polygoni Multiflori Radix30g	
**Lu (2016)**	Fuzheng decoction	Amomi Fructus 10g, Angelicae Sinensis Radix 20g, Angelicae Sinensis Radix 30g, Armeniacae Semen Amarum 12g, Astmgali Radix 30g, Coicis Semen 15g, Curcumae Rhizoma 15g, Fritillariae Thunbergii Bulbus 15g, Galli Gigerii Endothelium Corneum 15g, Glycyrrhizae Radix et Rhizoma Praeparata Cum Melle 10g, Hordei Fructus Germinatus 15g, Inulae Flos 15g, Magnoliae Officmalis Cortex 15g, Ophiopogonis Radix 20g, Paeoniae Radix Alba 15g, Pheretima 15g, Pinelliae Rhizoma 15g, Platycodonis Radix 15g, Poria 15g, Pseudostellariae Radix 20g, Rehmanniae Radix Praeparata 15g, Rubiae Radix et Rhizoma 15g, Spatholobi Caulis 30g	
**Wang (2016)**	Yangzhengxiaoji capsule	Astragali Radix, Ligustri lucidi Fructus, Ginseng Radix, Zedoariae Rhizoma, Ganoderma, Gynostemma pentaphylla, Atractylodis macrocephalae Rhizoma, Sculellaria barbata, Hedyotidis Diffusae Herba, Hoelen, Eupolyphaga sinensis, Galli Gigeriae Endothelium Corneum, duchesnea, Oriental wormwood, Cynanchi paniculati Radix	
**Xie (2016)**	Fuzheng Jiandu Kangai decoction	Fici Fructus 100 g, Coicis Semen 100 g, Rhizoma Imperatae100g, Liriopis Tuber 10 g, Pinelliae Tuber 15 g, Citri Unshii Pericarpium 3 g, Bambusae Caulis In Taeniam 12 g, Ginseng Radix 30 g, Fritillariae Thunbergii Bulbus 20 g, Glycyrrhizae Radix et Rhizoma 3g, Salviae Miltiorrhizae Radix 30 g, Schisandrae Fructus 10g, Notoginseng Radix 10 g, Amydae Carapax 20 g	
**Zhang (2016)**	Jiawei Baihe Gujin decoction	Rehmanniae Radix Crudus 10g, Rehmanniae Radix Preparata 12g, Liriopis Tuber 15g, Lilii Bulbus 20g, Bulbus Fritillariae Cirrhosae 9g, Scrophulariae Radix 10g, Adenophorae Radix 15g, Trichosanthis Radix 15,g Dendrobium nobile 12g	After radiation therapy, when there is no energy: biyan qingdu Keli: Panaciis Quinquefolii Radix 15g, Astragali Radix 15g; Afger radiation therapy, when throat is very dry: Adenophorae Radix 15g, Trichosanthis Radix 15g
	Biyan qing du Keli	n.r.	
**Sun (2017)**	Xufu zhuyu capsule	Persicae Semen, Carthami Flos, Paeoniae Radix Rubra, Cnidii Rhizoma, Aurantii Fructus Immaturus, Bupleuri Radix, Platycodi Radix, Angelicae Gigantis Radix, Rehmanniae Radix, Achyranthis Radix, Glycyrrhizae Radix et Rhizoma	
**Zhang (2017)**	Xihuang capsule	Calculus Bovis Artifactus, Artificial musk, Myrrha, Olibanum	

HM: herbal medicine; n.r.: not reported

**Table 3:** Herbs frequently used and common traditional Korean medicine diagnostic categories.

Herbal medicine	Frequency		TKM diagnosis
	Count	%	
Ophiopogonis Radix	12	52.1	Yin deficiency
Astragali Radix	10	43.5	Qi deficiency
Rehmanniae Radix Praeparata	9	39.1	Yin deficiency
Angelicae Sinensis Radix	8	34.8	Blood deficiency
Adenophorae Radix	7	30.4	Yin deficiency
Poria	7	30.4	Phlegm-retained fluid
Glycyrrhizae Radix et Rhizoma	7	30.4	Qi and Yin deficiency

TKM: traditional Korean Medicine

## Discussion

The present study reviewed 22 studies involving 1819 patients with lung cancer who received radiotherapy. The main finding of this study is that the number of patients who developed RP significantly decreased in the HM plus radiotherapy group compared with the radiotherapy alone group. In addition, the incidence rate of severe RP (scoring more than 3 points on the RTOG scale) decreased significantly in the groups that combined radiotherapy with HM when compared with radiotherapy alone. The KPS values, related to quality of life, also significantly increased in the HM plus radiotherapy group compared with the radiotherapy alone group. Although there were few studies that reported on the safety of HMs, no severe side effects of HMs were observed in those studies. However, due to problems related to the methodological quality and reporting in most of the trials, we are not able to draw definitive conclusions, and the results must be interpreted with caution.

The methodological quality and reporting of the trials were variable, and often inadequate. Most studies did not explain the randomization process they used, and the allocation method was not mentioned in a single study. Because it is difficult to use a placebo for herbal decoctions, the evaluation of patients after administration of herbal decoctions would be more valid if the assessors were also blinded. However, the assessors were not blinded in any of the studies. The quality of evidence for this finding was very low because of the high risk of bias. The quality of reporting was generally poor in the included studies (Table 4). Furthermore, since the number of subjects who were randomized or dropped out from the studies was unclear, it is difficult to tell whether the studies performed per-protocol analyses or intention-to-treat analyses. While it cannot be said, due to the low quality of reporting, that the clinical trials were not conducted properly, improvement in the quality of reporting is necessary, since quality assessments are based on reports.

**Table 4:** Summary of finding table.

Herbal Medicine compared to Radiotherapy for Radiation pneumonitis in Lung cancer						
**Patient or population:** patients with Radiation pneumonitis in Lung cancer **Settings:** **Intervention:** Herbal Medicine **Comparison:** Radiotherapy						
**Outcomes**	Illustrative comparative risks* **(95% CI)**		**Relative effect** **(95% CI)**	**No of Participants** **(studies)**	**Quality of the evidence** **(GRADE)**	**Comments**
	Assumed risk	Corresponding risk				
	**Radiotherapy**	**Herbal Medicine**				
**Incidence of radiation pneumonitis**	**357 per 1000**	**189 per 1000** (161 to 225)	**RR 0.53** (0.45 to 0.63)	1819 (22 studies)^a^	⊕⊝⊝⊝ **very low**^1^	
**Incidence of sever radiation pneumonitis**	**113 per 1000**	**25 per 1000** (12 to 46)	**RR 0.22** (0.11 to 0.41)	903 (11 studies)^b^	⊕⊝⊝⊝ **very low**^1^	
**Quality of life**		The mean quality of life in the intervention groups was **4.62 higher** (1.05 to 8.18 higher)		420 (6 studies)^c^	⊕⊝⊝⊝ **very low**^1^	

*The basis for the **assumed risk** (e.g. the median control group risk across studies) is provided in footnotes. The **corresponding risk** (and its 95% confidence interval) is based on the assumed risk in the comparison group and the **relative effect** of the intervention (and its 95% CI).

**CI:** Confidence interval; **RR:** Risk ratio;

GRADE Working Group grades of evidence

**High quality:** Further research is very unlikely to change our confidence in the estimate of effect.

**Moderate quality:** Further research is likely to have an important impact on our confidence in the estimate of effect and may change the estimate.

**Low quality:** Further research is very likely to have an important impact on our confidence in the estimate of effect and is likely to change the estimate.

**Very low quality:** We are very uncertain about the estimate.

^a^ Cao (2013), Du (2013), Fu (2008), Gao (2012), Jiang (2011), LI (2014), Lu (2016), Meng (2012), Song (2007), Sun (2017), Tang (2009), Wang (2006), Wang (2011), Wang (2016), Xiao (2010), Xie (2016), Xu (2013), Yin (2013), Zhang (2006), Zhang (2016), Zhang (2017), Zhou (2009)

^b^ Cao (2013), Du (2013), Gao (2012), Li (2014), Lu (2016), Meng (2012), Wang (2006), Xie (2016), Zhang (2006)

^c^ Du (2013), Li (2014), Meng (2012), Tang (2009), Xiao (2010), Xie (2016)

The chemical components of HMs can change according to the cultivation region, climate, and cultivation season. Therefore, clinical studies must provide detailed information about the HMs used. The Consolidated Standards of Reporting Trials (CONSORT) provides guidelines that list information requirements regarding HMs that must be met by clinical studies to improve the reporting quality of RCTs [43]. Recently CONSORT extensions for Chinese herbal medicine (CHM) formulas (CONSORT-CHM Formulas 2017) was developed to offer more suitable guideline for HM formulas [44]. Traditional Chinese medicine (TCM) and traditional Korean medicine (TKM) is based on its own unique principle and comprehensive theory. The CONSORT—CHM Formulas 2017 reported the key concepts of pattern and the features of CHM formulas to reflect TCM- theory. The selected studies provided poor information on the HMs that they investigated. None of the studies reported herbal medicinal product names, characteristics of the herbal products, dosage regimen and quantitative description, or qualitative testing, which should have been explained. The selected studies only reported the amount of each herb constituting the HM in grams, and the daily dosage. Future clinical studies using HMs must be conducted with more care and recommended to follow the CONSORT—CHM Formulas 2017.

Many of the studies used the RTOG scale, which is an appropriate assessment tool for RP, used internationally. The quality of life was evaluated using the KPS scale; however, strictly speaking, the KPS scale is a performance scale, not an assessment tool. The quality of life needs to be evaluated with internationally validated assessment tools, such as the EORTC-C30 [45] and EORTC-L13 [46] or fact-L questionnaires [47] developed for patients with lung cancer.

The prescriptions mainly comprised herbs that nourish yin, tonify qi and nourish blood. *Ophiopogonis Radix*, *Astragali Radix*, and *Angelicae Sinensis Radix* have been reported to be frequently used in traditional Chinese medicine in combination with chemotherapy for patients with non-small cell carcinoma [48]. *Astragali Radix*, *Adenophorae Radix*, *Ophiopogonis Radix*, *Glycyrrhizae Radix et Rhizoma*, and *Poria* are in line with the frequently used herbs as adjuvant therapy to chemotherapy in patients with non-small cell carcinoma [15]. As shown here, the herbal formulas used with radiotherapy mainly comprised herbs that nourish yin, coupled with qi-tonifying herbs. The proportion of yin-nourishing herbs was higher than in formulas used for chemotherapy. This is because radiation therapy is regarded as a heat toxin pathogen in traditional Chinese and Korean medicine theory, so prescriptions generally focus on removing heat. Despite the limitation that the herbal formulas covered in this review were not consistent, it is promising that co-administration lowered the incidence of radiation pneumonia. Future studies could develop and verify mixed herbal medicines with the most active ingredients.

This study had the following strengths. First, 11 databases were searched using a well-structured search formula, and were selected through proper processes. We tried to minimize bias in the assessment of the effects of HMs on the incidence rate of RP, which was the primary goal of this study, by using studies that accurately described the diagnostic criteria of RP. Second, studies from all the countries in which RCTs using HMs have been actively conducted were included, including English-speaking countries, China, and Korea. The primary limitation of this study was that the quality of reporting was low in the selected studies, making clear interpretation of the results difficult.

To the best of our knowledge, this is the first systematic review that investigates the efficacy and safety of orally administered HM in conjunction with radiotherapy for preventing radiation induced pneumonitis in patients with lung cancer. The present systemic review showed that administration of HMs during radiotherapy could prevent or minimize the risk of radiotherapy pneumonia. However, due to the poor methodological quality of the identified studies, definitive conclusion could not be drawn. Randomized controlled trials that are larger in scale and have better designs must be conducted to confirm the validity of this finding.

## Supporting information

S1 Appendix Search strategies. (DOCX)

S2 Appendix Reference of the excluded studies. (XLSX)

S3 Appendix PRISMA checklist. (DOC)
