# Supplementary material for: Oral administration of herbal medicines for radiation pneumonitis in lung cancer patients: A systematic review and meta-analysis
Source: PLoS One. 2018 May 30;13(5):e0198015. doi: 10.1371/journal.pone.0198015 (PMC5976163; doi:10.1371/journal.pone.0198015)
Supplement: S1 Appendix — (DOCX) [file pone.0198015.s001.docx]

**S1 Appendix. Search strategies**

**Table 1 The Cochrane Library**

| #1 | MeSH descriptor: [Radiation Pneumonitis] explode all trees |
| --- | --- |
| #2 | "radiation pneumonia" in All Text |
| #3 | “radiation pneumonias” in All Text |
| #4 | "radiation pneumonitis" in All Text |
| #5 | "radiation pneumonitides" in All Text |
| #6 | (radiation near/3 pneumoni*) in All Text |
| #7 | #1 or #2 or #3 or #4 or #5 or #6 |
| #8 | MeSH descriptor: [Medicine, African Traditional] explode all trees |
| #9 | MeSH descriptor: [Medicine, Arabic] explode all trees |
| #10 | MeSH descriptor: [Medicine, Ayurvedic] explode all trees |
| #11 | MeSH descriptor: [Medicine, Traditional] explode all trees |
| #12 | MeSH descriptor: [Medicine, East Asian Traditional] explode all trees |
| #13 | MeSH descriptor: [Medicine, Korean Traditional] explode all trees |
| #14 | MeSH descriptor: [Medicine, Kampo] explode all trees |
| #15 | MeSH descriptor: [Medicine, Chinese Traditional] explode all trees |
| #16 | MeSH descriptor: [Medicine, Tibetan Traditional] explode all trees |
| #17 | MeSH descriptor: [Drugs, Chinese Herbal] explode all trees |
| #18 | MeSH descriptor: [Plants] explode all trees |
| #19 | MeSH descriptor: [Plants, Medicinal] explode all trees |
| #20 | MeSH descriptor: [Phytotherapy] explode all trees |
| #21 | MeSH descriptor: [Plant Extracts] explode all trees |
| #22 | MeSH descriptor: [Plant Preparations] explode all trees |
| #23 | MeSH descriptor: [Ethnobotany] explode all trees |
| #24 | herb* OR plant* OR plant* extract |
| #25 | #8 or #9 or #10 or #11 or #12 or #13 or #14 or #15 or #16 or #17 or #18 or #19 or #20 or #21 or #22 or #23 or #24 |
| #26 | #7 and #25 |

**Table 2 Medline (PubMed)**

| #1 | Radiation pneumonitis[mh] |
| --- | --- |
| #2 | Pneumonitis, radiation |
| #3 | Radiation Pneumonitides |
| #4 | Pneumonitides, radiation |
| #5 | Radiation pneumonia |
| #6 | Pneumonia, radiation |
| #7 | Radiation pneumonias |
| #8 | Pneumonias, radiation |
| #9 | #1 or #2 or #3 or #4 or #5 or #6 or #7 or #8 |
| #10 | Medicine, African traditional[mh] |
| #11 | Medicine, Arabic[mh] |
| #12 | Medicine, ayurvedic[mh] |
| #13 | Medicine, traditional[mh] |
| #14 | Medicine, east Asian traditional[mh] |
| #15 | Medicine, Korean traditional[mh] |
| #16 | Medicine, Kampo[mh] |
| #17 | Medicine, Chinese traditional[mh] |
| #18 | Medicine, Tibetan traditional[mh] |
| #19 | Drugs, Chinese herbal[mh] |
| #20 | Herb, medicinal[mh] |
| #21 | Herbal therapy[mh] |
| #22 | Medicinal plants[mh] |
| #23 | Plant extracts[mh] |
| #24 | Phytotherapy[mh] |
| #25 | Plants[mh] |
| #26 | Plant preparations[mh] |
| #27 | Ethnobotany[mh] |
| #28 | Herb*[tiab] |
| #29 | #10 or #11 or #12 or #13 or #14 or #15 or #16 or #17 or #18 or #19 or #20 or #21 or #22 or #23 or #24 or #25 or #26 or #27 or #28 |
| #30 | Randomized controlled trial[pt] |
| #31 | Controlled clinical trial[pt] |
| #32 | Randomized[tiab] |
| #33 | Placebo[tiab] |
| #34 | Randomly[tiab] |
| #35 | Trial[tiab] |
| #36 | Groups[tiab] |
| #37 | #30 or #31 or #32 or #33 or #34 or #35 or #36 |
| #38 | #9 and #29 and #37 |

**Table 3 EMBASE**

| #1 | 'radiation pneumonia'/exp OR 'radiation pneumonia' |
| --- | --- |
| #2 | (radiation NEAR/3 pneumoni*):ab,ti |
| #3 | ‘radiation pneumonitides’ |
| #4 | #1 or #2 or #3 |
| #5 | 'traditional medicine'/exp |
| #6 | 'african medicine'/exp |
| #7 | ‘arabic medicine’ |
| #8 | ‘ayurveda’/exp |
| #9 | 'korean medicine'/exp |
| #10 | 'oriental medicine'/exp |
| #11 | 'chinese medicine'/exp |
| #12 | ‘tibetan medicine’/exp |
| #13 | 'kampo'/exp |
| #14 | 'herbal medicine'/exp |
| #15 | 'herbaceous agent'/exp |
| #16 | 'medicinal plant'/exp |
| #17 | 'plant'/exp |
| #18 | 'plant extract'/exp |
| #19 | ‘plant medicinal product’/exp |
| #20 | 'phytotherapy'/exp |
| #21 | ‘herb’/exp |
| #22 | 'ethnobotany'/exp |
| #23 | #5 or #6 or #7 or #8 or #9 or #10 or #11 or #12 or #13 or #14 or #15 or #16 or #17 or #18 or #19 or #20 or #21 or #22 |
| #24 | 'clinical trial'/exp OR 'clinical trial' |
| #25 | ‘controlled clinical trial’/exp |
| #26 | ‘randomization’/exp |
| #27 | ‘double blind procedure’/exp OR ‘double blind procedure’ |
| #28 | ‘single blind procedure’/exp OR ‘single blind procedure’ |
| #29 | ‘placebo’/exp |
| #30 | ‘prospective’/exp |
| #31 | group:ab |
| #32 | #24 or #25 or #26 or #27 or #28 or #29 or #30 or #31 |
| #33 | #4 and #23 and #32 |

**Table 4 CNKI**

| 1 | Pneumonitides, radiation |
| --- | --- |
| 2 | Radiation pneumonitides |
| 3 | Radiation pneumonitis |
| 4 | Radiation pneumonia |
| 5 | Radiation pneumonias |
| 6 | 发放射性肺炎 |
| 7 | 放射性肺炎 |
| 8 | acute radiation pneumonitis |
| 9 | 急性放射性肺炎 |
| 10 | 1 or 2 or 3 or 4 or 5 or 6 or 7 or 8 or 9 |
| 11 | 中药 |
| 12 | 胶囊 |
| 13 | 散 |
| 14 | 汤 |
| 15 | 颗粒 |
| 16 | 自拟剂 |
| 17 | Herbal medicine |
| 18 | Chinese medicine |
| 19 | Capsule |
| 20 | herb |
| 21 | decoction |
| 22 | 中西医 |
| 23 | 中医药 |
| 24 | plants |
| 25 | 11 or 12 or 13 or 14 or 15 or 16 or 17 or 18 or 19 or 20 or 21 or 22 or 23 or 24 |
| 26 | 临床研究 |
| 27 | 临床观察 |
| 28 | 随机 |
| 29 | 随机对照 |
| 30 | RCT |
| 31 | Randomized controlled trials |
| 32 | 26 or 27 or 28 or 29 or 30 or 31 |
| 33 | 10 and 25 and 32 |
